# Numerical dataset for analyzing the performance of a highly efficient ultrathin film CdTe solar cell

**DOI:** 10.1016/j.dib.2017.04.015

**Published:** 2017-04-19

**Authors:** Rucksana Safa Sultana, Ali Newaz Bahar, Md. Asaduzzaman, Mohammad Maksudur Rahman Bhuiyan, Kawsar Ahmed

**Affiliations:** a Department of Information and Communication Technology (ICT), Mawlana Bhashani Science and Technology University (MBSTU), Santosh, Tangail 1902, Bangladesh; b University Grants Commission of Bangladesh, Dhaka, Bangladesh

**Keywords:** CdTe, Photovoltaic cell, Efficiency, Layer properties, Numerical modeling

## Abstract

The article comprises numerical data of distinct semiconductor materials applied in the sketch of a CdTe absorber based ultrathin film solar cell. Additionally, the contact layer parametric values of the cell have been described also. Therefore, the simulation has been conducted with data related to the hetero-structured (n-ZnO/n-CdS/p-CdTe/p-ZnTe) semiconductor device and a *J*–*V* characteristics curve was obtained. The operating conditions have also been recorded. Afterward, the solar cell performance parameters such as open circuit voltage (*V*_oc_), short circuit current density (*J*_sc_), fill factor (FF), and efficiency (*η*) have been investigated and compared with reference cell.

**Specifications Table**

**Table:** 

Subject area	*Physics*
More specific subject area	*Solar energy*
Type of data	*Figures and tables*
How data was acquired	*Physical data were acquired from Refs.*[1], [2], [3], [4], [5], [6], [7], [8], [9]*and the performance parameter dataset has been simulated by ADEPT 2.1*[10].
Data format	*Filtered and analyzed*
Experimental features	*A CdTe solar cell has been organized as n-ZnO/n-CdS/p-CdTe/p-ZnTe heterojunction. Therefore, based on the effects of layer thickness, band gap, doping concentration, refractive index, and others electrical and mechanical properties of the materials, the quantities of the performance parameters have been examined.*
Data accessibility	*Data are available inside the article*

**Value of the data**

• The physical dataset provides basic standard data for simulating a CdTe ultrathin film photovoltaic cell.
• Researchers can go forward the theoretical analysis of a solar cell utilizing the same dataset
• Included dataset assists to compare and authorize the theoretical results of other models and approaches.
• Dataset contributes to an elaboration of knowledge and finds new concept for CdTe cell analysis.
• The performance parameter dataset can be used to equate the future simulation in CdTe solar cell technology.

## 1 Data

**Fig. 1:**
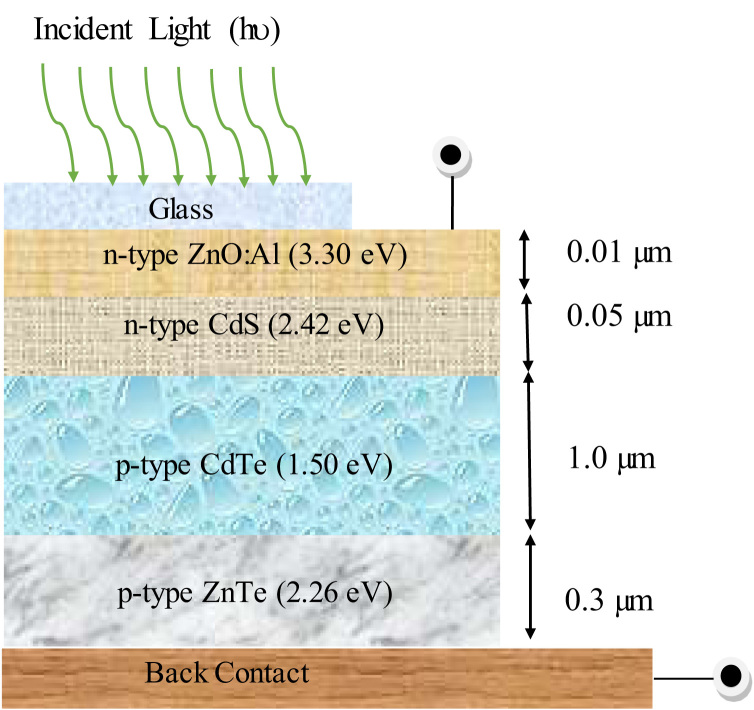
Schematic design of CdTe solar cell.

**Fig. 2:**
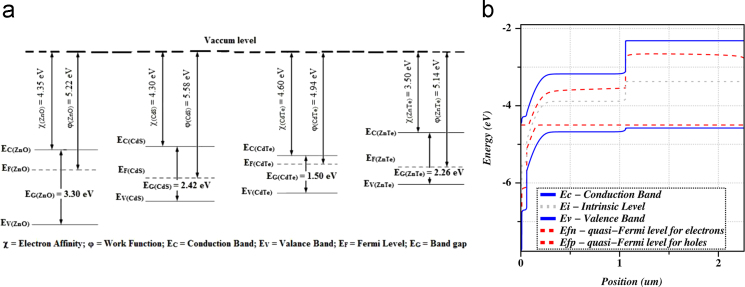
(a) Energy band diagram using Anderson׳s electron affinity rule (Vacuum level as reference level); (b) Energy band diagram (Fermi level as reference level).

This paper presents the numerical data for sketching a highly efficient CdTe solar cell. The baseline data of different layers used for simulation have been presented in Table 1. The contact parameters have also been listed in Table 2. Fig. 1 represents the schematic diagram for CdS/CdTe/ZnTe photovoltaic cell. The simulation was conducted under some conditions that have been evidenced on Table 3. ADEPT simulator supplies illumination estimating natural sunlight. All the dataset has been acquired from the issued research articles [1], [2], [3], [4], [5], [6], [7], [8], [9], [10]. Fig. 2 shows the *J*–*V* characteristics curve. Table 4 describes the comparison between performance measurement parameters of the optimized and reference CdTe photovoltaic cell.

## 2 Experimental design, materials and methods

### 2.1 Cell structure of CdTe solar cell

The schematic design for CdTe solar cell has been visualized in Fig. 1. It consists of n-ZnO buffer/n-CdS window/p-CdTe absorber/p-ZnTe back surface field (BSF) layer with glass at the front contact and metal back contact. The simulation was conducted under AM1.5G illumination to explain the incident sunlight. Fig. 2(a) describes the energy band diagram using Anderson׳s electron affinity rule where the vacuum level has been used as the reference level. In Fig. 2(b), the Fermi level has been used as the reference level of the energy band diagram.

### 2.2 Performance measurement of CdTe solar cell

**Fig. 3:**
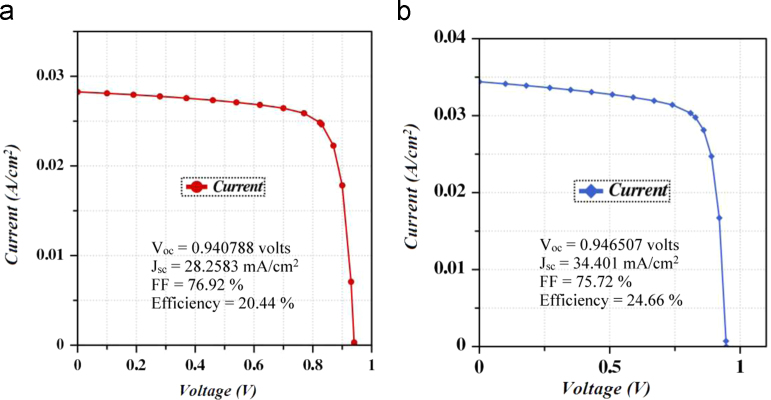
(a) *J*–*V* characteristic curve for CdTe solar cell (without BSF layer); (b) *J*–*V* characteristic curve for CdTe solar cell (with BSF layer).

A one-dimensional simulation software, ADEPT/F 2.1 is employed to simulate the electrical characteristics of hetero-structured semiconductor device [11]. The optimized values of open circuit voltage (*V*_oc_) and short circuit current (*J*_sc_) for CdTe solar cell without BSF layer and with BSF layer have been measured from the *J*–*V* characteristic curve as depicted in Fig. 3(a) and (b) respectively. Accordingly, the optimum FF and *η* have been found out from the simulation result of the CdTe cell. All the data values reporting the performance of the optimized CdTe cell with respect to the reference cell are demonstrated in Table 4.

## Figures and Tables

**Table 1:** Baseline data for different layers used for modeling CdTe solar cell.

**Parameters**	**n-ZnO: Al**	**n-CdS**	**p-CdTe**	**p-ZnTe**
Thickness, *t*_m_ (µm)	0.01	0.05	1.00	0.30
Band gap, *E*_g_ (eV)	3.30	2.42	1.50	2.26
Dielectric constant, *K*_S_	9.00	10.00	9.40	9.67
Electron affinity, *χ*_e_ (eV)	4.35	4.30	4.60	3.50
Refractive index, *N*_dx_	2.00	3.15	3.67	4.00
Electron mobility, *µ*_n_ (cm^2^ V^−1^ s^−1^)	100	100	320	330
Hole mobility, *µ*_p_ (cm^2^ V^−1^ s^−1^)	25	25	40	80
Effective mass for electrons, *m*_n_*/*m*_0_	0.27	0.17	0.25	0.13
Effective mass for holes, *m*_p_*/*m*_0_	0.59	0.70	0.70	0.60
Conduction band effective density of states, *N*_C_ (cm^−3^)	2.2×10^18^	2.2×10^17^	8×10^17^	7×10^16^
Valence band effective density of states, *N*_V_ (cm^−3^)	1.8×10^19^	1.8×10^18^	1.8×10^19^	2×10^19^
Donor concentration, *N*_d_ (cm^−3^)	1×10^18^	1×10^17^	–	–
Acceptor concentration, *N*_a_ (cm^−3^)	–	–	2×10^16^	1×10^18^
Electron capture cross section, *σ*_e_ (cm^2^)	1×10^−12^	1×10^−17^	1×10^−11^	1×10^−11^
Hole capture cross section, *σ*_h_ (cm^2^)	1×10^−15^	1×10^−12^	1×10^−14^	1×10^−16^
Electron lifetime, *τ*_n_ (s)	5×10^−8^	2×10^−8^	1×10^−8^	1×10^−5^
Hole lifetime, *τ*_p_ (s)	5×10^−9^	6×10^−8^	5×10^−8^	1×10^−4^
Standard deviation, *σ*_d_ (eV)	0.1	0.1	0.1	0.1

**Table 2:** Contact layer data used for modeling CdTe solar cell.

**Parameters**	**Front contact**	**Back contact**
Barrier height, *φ*_b_ (eV)	*Ф*_bn_=0.03	*Ф*_bp_=1.90
Reflectance, *R*_f_ (I)	0.2	0.8
Recombination velocity for holes, *S*_h_ (cm s^−1^)	1×10^7^	1×10^7^
Recombination velocity for electrons, *S*_e_ (cm s^−1^)	1×10^7^	1×10^7^

**Table 3:** Operating conditions based on which the simulation was carried out.

Operating conditions	Description
Terrestrial illumination	AM1.5G
Solar irradiance on earth, *E* (W cm^−2^)	0.1
Temperature, *T*_K_ (k)	300
Shadowing factor	0.02

**Table 4:** Comparison between optimized performance parameters of simulated and reference CdTe cell [12].

Cells	Open circuit voltage, *V*_oc_ (mV)	Short circuit current density, *J*_sc_ (mA/cm^2^)	Fill factor, FF (%)	Efficiency, *η* (%)
Reference cell [11]	887.20	31.69	78.50	22.10
CdTe cell without BSF layer	940.79	28.26	76.92	20.44
CdTe cell with BSF layer	946.51	34.40	75.72	24.66
